# Size-dependent changes in wood chemical traits: a comparison of neotropical saplings and large trees

**DOI:** 10.1093/aobpla/plt039

**Published:** 2013-08-29

**Authors:** Adam R. Martin, Sean C. Thomas, Yong Zhao

**Affiliations:** Faculty of Forestry, University of Toronto, Earth Sciences Building, 33 Willcocks Street, Toronto, ON, CanadaM5S 3B3

**Keywords:** Functional traits, pathogens, phylogenetic analysis, plant defence, resource availability hypothesis, tropical forest, tropical tree, wood economics, wood traits.

## Abstract

Wood anatomical traits are important correlates of life-history strategies among tree species, yet little is known about wood chemical traits. Additionally, size-dependent changes in wood chemical traits have been rarely examined, although these changes may represent an important aspect of tree ontogeny. Owing to selection for pathogen resistance and biomechanical stability, we predicted that saplings would show higher lignin (L) and wood carbon (C_conv_), and lower holocellulose (H) concentrations, compared with conspecific large trees. To test these expectations, we quantified H, L and C_conv_ in co-occurring Panamanian tree species at the large tree vs. sapling size classes. We also examined inter- and intraspecific patterns using multivariate and phylogenetic analyses. In 15 of 16 species, sapling L concentration was higher than that in conspecific large trees, and in all 16 species, sapling H was lower than that in conspecific large trees. In 16 of 24 species, C_conv_ was higher in saplings than conspecific large trees. All large-tree traits were unrelated to sapling values and were unrelated to four life-history variables. Wood chemical traits did not show a phylogenetic signal in saplings, instead showing similar values across distantly related taxa; in large trees, only H showed a significant phylogenetic signal. Size-dependent changes in wood chemistry show consistent and predictable patterns, suggesting that ontogenetic changes in wood chemical traits are an important aspect of tree functional biology. Our results are consistent with the hypothesis that at early ontogenetic stages, trees are selected for greater L to defend against cellulose-decaying pathogens, or possibly to confer biomechanical stability.

## Abstract (precis)

We have a fundamental and applied understanding of how differences in the wood chemistry of trees affects the durability of wood products. By comparison, relatively little is known about the ecological causes and consequences of species differences in wood chemistry; even less is known about how or why wood chemistry differs within species, across trees of different sizes. In this study we find strong and consistent differences in wood chemistry of saplings and canopy trees, in several tropical hardwood species. These differences point to the importance of pathogens and tree biomechanics as evolutionary causes of size-dependent changes in wood chemistry.

## Introduction

In recent years, certain wood functional traits, namely wood density (WD), xylem vessel anatomy and wood carbon (C) concentration, have become of considerable interest to ecologists and plant biologists. At the species level, this interest has been promoted by numerous studies finding significant correlations between wood traits and tree demographic rates (e.g. Poorter *et al.* 2008; Wright *et al.* 2010), and other tree life-history characteristics such as maximum tree size and leaf physiology and morphology (reviewed by Chave *et al.* 2009). At the ecosystem level of integration, these wood traits have been found to be important correlates of functional characteristics such as forest C storage (Baker *et al.* 2004; Martin and Thomas 2011; Thomas and Martin 2012) and nutrient cycling associated with wood decomposition (Weedon *et al.* 2009; van Geffen *et al.* 2010).

Yet important aspects of wood functional traits—hypothesized to covary along a ‘wood economics spectrum’ (WES; Chave *et al.* 2009)—still remain poorly understood when compared with other notable suites of functional traits such as leaf traits, reproductive traits or tree size metrics (reviewed by Westoby *et al.* 2002). For tropical trees, this is largely because wood chemical traits have received very little attention, in contrast to anatomical traits. For example, large databases currently contain ∼6200 species-specific WD values for tropical trees (Zanne *et al.* 2009), but <150 species-specific records for wood C in tropical hardwoods (Thomas and Martin 2012).

For tropical woods, even less data are readily available for lignin (L) and holocellulose (H) concentrations—the latter defined as total cellulose and hemicellulose concentration—which are the most abundant chemical compounds in wood, comprising ∼20–35 and 65–75 % of woody tissues on a dry mass basis, respectively (Pettersen 1984). Lignin and H have been identified as traits potentially contributing to the WES (Chave *et al.* 2009), largely due to their role in crucial ecological functions such as stem resistance to damage from insects and pathogens (Pew 1957; Scheffer and Cowling 1966; Wainhouse *et al.* 1990; Kurokawa *et al.* 2004), stem mechanical stability (Whetten and Sederoff 1995; Alvarez-Clare and Kitajima 2007) and wood decomposition (Cornwell *et al.* 2009; Weedon *et al.* 2009; van Geffen *et al.* 2010). While several studies have examined L and H in tropical trees in relation to the production and durability of wood products, surprisingly few have examined the ecological significance of H and L. For tropical trees, only two studies have quantified the relationship between wood L and species' demography and these results were mixed. Stem L was not correlated with seedling survival in eight Panamanian tree species (Alvarez-Clare and Kitajima 2007), but a reference to unpublished data in Kurokawa *et al.* (2004) suggested that L was positively associated with seedling survivorship in three Bornean tree species.

Holocellulose and L might be expected to show relationships with species' life-history traits and show strong evolutionary patterns. In addition to its biomechanical function, there is strong evidence for a defensive role of lignin (Vance *et al.* 1980), and L may thus be considered a measure of defensive investment. But because L is a more energetically expensive compound (∼60–72 % C) than cellulose or other compounds such as non-structural carbohydrates (∼40–44 % C; Pettersen 1984), any putative pest/pathogen resistance conferred by L may come at the expense of plant growth. Under this assumption, L (and by association H) would be expected to correlate with species' demographic rates. Additionally, since demographic rates are often phylogenetically conserved (Kraft *et al.* 2010), H and L could also be expected to be more similar among closely related taxa than expected by chance alone (i.e. show a significant phylogenetic signal).

An additional aspect of wood chemical traits in tropical trees that remains poorly resolved is the potential occurrence of size-dependent variation. Virtually all evidence of size-dependent changes in wood traits comes from studies documenting within-stem trait variation (i.e. variation occurring from pith to bark within a tree, e.g. Lamlom and Savidge 2006; Lachenbruch *et al.* 2011). Studies explicitly testing for size-dependent changes in wood chemical traits of trees at distinct life-history stages remain scarce, despite good reason to expect such ontogenetic changes.

Studies point to a greater demand for L investment as a defensive compound in seedling and sapling wood, when compared with later ontogenetic stages. Generalist soil-borne fungal pathogens that degrade cellulose (i.e. ‘damping-off’ diseases in the genus *Pythium*) may account for ≥75 % of seedling and sapling mortality in some tropical tree species, largely by inducing xylem dysfunction (Augspurger and Kelly 1984; Augspurger and Wilkinson 2007). However, when ligno-cellulosic complexes are present, the spread of cellulose-degrading pathogens in wood is slowed (Pew 1957), suggesting that higher L concentrations in seedlings could decrease the susceptibility to damping-off disease, and thereby enhance survival in the forest understorey. Beyond the smaller tree sizes (i.e. ≥1 cm diameter at breast height; DBH), cellulose-degrading pathogens *per se* become less important as agents of tree mortality, and are not common on live trees (Gilbert *et al.* 2002). Following this reasoning, we expect that saplings should show higher L on a weight/weight basis (and lower H) when compared with conspecific large trees, due to greater need for chemical compounds that confer pathogen resistance.

Size-dependent changes in L (and by association H) could also possibly be due to changing requirements for mechanical stability. Higher wood L concentrations are correlated with greater wood stiffness (Niklas 1992; Alvarez-Clare and Kitajima 2007; Voelker *et al.* 2011), though not necessarily other mechanical properties, as L in isolation has low mechanical strength (Winandy and Rowell 2005). Resistance to wind and stem buckling increases with tree size largely due to increases in trunk diameter (Sterck and Bongers 1998) and root buttressing (Smith 1972), factors that are limited in seedlings and saplings. Therefore, seedlings and saplings may rely on greater L concentrations (and lower H concentrations) to maintain stem rigidity: a developmental constraint leading to consistent ontogenetic changes in wood chemistry across species.

A corollary of expected size-dependent declines in L concentrations is that saplings should have higher wood C concentrations compared with conspecific larger trees, since L is more carbon rich compared with other chemical compounds such as cellulose (Pettersen 1984). Interestingly, this expectation is counter to prominent forest C accounting protocols (IPCC 2006), which assume that wood in small tropical trees (<10 cm DBH) is less C rich (46 % C w/w) when compared with woody biomass in larger tropical trees (≥10 cm DBH containing 49 % C w/w). The IPCC (2006) based these protocols on a single study (Hughes *et al.* 2000) that lacked explicit comparisons between size categories within any tree species.

In the present study, we sought to increase our understanding of wood traits in tropical trees by comparing wood chemical traits of saplings and large trees in 24 Panamanian rainforest tree species. We sought to answer the following questions: (i) Does wood from saplings have higher L and C concentrations, and lower H concentration, than that in conspecific large trees? (ii) Are wood chemical traits in large tropical trees predicted by conspecific sapling traits? (iii) Are wood H and L concentration related to life-history strategies in tropical trees? (iv) Are wood chemical traits similar among closely related taxa in either saplings or large trees?

## Methods

### Study site and sample collection

We collected wood samples from Soberania National Park (SNP), an ∼22 000-ha tract of semi-deciduous lowland moist forest located in central Panama (9°10′N, 75°45′W). Our study was restricted to the Pipeline Road site, where forests are second growth, and range from 30 to 200 m in elevation, with canopy heights of ∼20–40 m (Martin and Thomas 2011). Forests in SNP experience a tropical monsoon climate, receiving average rainfalls of ∼2100 mm year^−1^ and mean monthly temperatures of ∼27 °C (Croat 1978). The forests are seasonal, with a 4-month dry season occurring December through April, during which <10 % of total annual precipitation falls (Croat 1978).

In August 2008, we selected three to five individuals (depending on the presence of suitable stems, see below) from 24 tree species (Table 1) from each of the two size classes: saplings (≤1 m and ≥25 cm in height) and large trees (≥10 cm DBH). Standardizing large-tree sizes within a more specific DBH range was logistically intractable at our study site due to high species diversity. Species included in the study were common at the sapling stage in the SNP understorey, and common at larger sizes in both SNP and the nearby (<15 km) Barro Colorado Island (BCI) forest dynamics plot (9°15′N, 79°85′W). At BCI, our study species accounted for 9.4 % of individual stems and 16.2 % of tree basal area, for trees sizes ≥1 cm DBH during a 2000–2005 census interval (Hubbell *et al.* 2005). Species were also chosen to provide a broad taxonomic range (Table 1), and to represent a range of life-history strategies from light-demanding pioneer species, to shade-tolerant late-successional species.

**Table 1.** Wood chemical traits for 24 Panamanian tree species. Taxonomy, holocellulose content (H), lignin content (L), cellulose : lignin ratios (H : L) and carbon content (C_conv_) for Panamanian rainforest tree species at the sapling (subscript ‘sap’) and large-tree (subscript ‘large’) size groups. Superscripts following species names refer to: ^a^H_large_, L_large_ and H : L_large_ data from Pettersen (1984); ^b^H_large_, L_large_ and H : L_large_ data are means for *n* = 2 samples; ^c^H_large_, L_large_ and H : L_large_ data are based on *n* = 1 sample; ^d^C_conv-large_ data taken from Martin and Thomas (2011).

Species	Holocellulose (H)		Lignin (L)		Cellulose : lignin (H : L)		Carbon (C_conv_)	
	H_sap_	H_large_	L_sap_	L_large_	H : L_sap_	H : L_large_	C_conv-sap_	C_conv-large_
*Alseis blackiana* (Rubiaceae)	56.4 ± 0.7	64.3 ± 0.8	34.3 ± 2.5	29.2 ± 1.5	1.7 ± 0.1	2.2 ± 0.1	53.2 ± 0.1	49.2 ± 1.5
*Anacardium excelsum* (Anacardiaceae)^a^	53.5 ± 1.3	72.0	39.8 ± 1.5	27.0	1.4 ± 0.03	2.7	48.9 ± 0.5	47.3 ± 1.3
*Cinnamomum triplinerve* (Lauraceae)^b^	60.1 ± 0.2	66.6 ± 0.1	32.4 ± 1.5	31.8 ± 2.2	1.9 ± 0.1	2.1 ± 0.2	49.0 ± 0.8	48.7 ± 1.6
*Cupania latifolia* (Sapindaceae)	58.1 ± 1.2	74.9 ± 3.7	37.7 ± 1.1	25.1 ± 1.5	1.5 ± 0.02	3.0 ± 0.1	47.9 ± 1.2	49.5 ± 0.3
*Cupania rufescens* (Sapindaceae)	55.6 ± 0.8	72.9 ± 1.2	32.9 ± 2.1	27.1 ± 1.9	1.7 ± 0.1	2.7 ± 0.3	50.6 ± 0.6	50.4 ± 0.7
*Guarea guidonia* (Meliaceae)	52. 5 ± 1.2	67.6 ± 2.0	45.9 ± 1.7	27.2 ± 0.3	1.1 ± 0.1	2.5 ± 0.1	47.2 ± 1.5	49.0 ± 0.4
*Gustavia superba* (Lecythidaceae)	53.8 ± 1.0	64.0 ± 1.1	42.7 ± 1.7	36.1 ± 1.1	1.3 ± 0.1	1.8 ± 0.1	46.2 ± 1.3	45.9 ± 0.6
*Poulsenia armata* (Moraceae)^a^	54.1 ± 1.0	64.0	38.6 ± 0.4	36.0	1.4 ± 0.03	1.8	45.1 ± 0.8	46.5 ± 1.5
*Protium costaricense* (Burseraceae)	56.6 ± 1.0	71.1 ± 1.1	43.4 ± 0.9	23.8 ± 1.4	1.3 ± 0.03	3.0 ± 0.2	50.9 ± 1.0	48.5 ± 0.7
*Protium tenuifolium* (Burseraceae)	60.7 ± 1.9	73.1 ± 2.1	30.8 ± 0.9	26.9 ± 0.2	2.0 ± 0.1	2.7 ± 0.1	49.8 ± 1.1	49.1 ± 0.3
*Pseudobombax septenatum* (Malvaceae)^c^	53.6 ± 2.3	73.0	40.5 ± 1.1	21.3	1.3 ± 0.1	3.4	50.2 ± 0.5	45.4 ± 0.8
*Sapium glandulosum* (Euphorbiaceae)	59.9 ± 1.0	69.0 ± 1.2	40.2 ± 2.5	27.9 ± 0.1	1.5 ± 0.1	2.5 ± 0.1	46.5 ± 1.6	47.9 ± 0.1
*Trichilia pallida* (Meliaceae)	59.6 ± 1.5	71.3 ± 0. 5	40.4 ± 1.1	17.2 ± 0.8	1.5 ± 0.04	4.2 ± 0.2	50.5 ± 2.0	50.4 ± 0.3
*Virola multiflora* (Myristicaceae)^b^	62.2 ± 0.7	64.5 ± 1.3	35.3 ± 2.3	35.5 ± 2.7	1.8 ± 0.1	1.8 ± 0.2	49.6 ± 0.6	48.8 ± 0.2
*Virola sebifera* (Myristicaceae)	58.7 ± 1.6	68.0 ± 0.3	32.0 ± 0.8	24.4 ± 1.3	1.8 ± 0.04	2.8 ± 0.1	52.7 ± 1.0	48.9 ± 1.2
*Zanthoxylum ekmanii* (Rutaceae)	60.1 ± 2.0	71.9 ± 1.7	36.4 ± 0.7	26.3 ± 2.4	1.7 ± 0.1	2.7 ± 0.2	49.4 ± 0.6	50.7 ± 0.6
*Annona spraguei* (Annonaceae)	NA	NA	NA	NA	NA	NA	50.6 ± 1.1	45.2 ± 0.2
*Croton billbergianus* (Euphorbiaceae)	NA	NA	NA	NA	NA	NA	47.8 ± 0.7	44.5 ± 0.4
*Guarea ‘fuzzy’* (Meliaceae)	NA	NA	NA	NA	NA	NA	49.8 ± 0.9	43.2 ± 0.1
*Hieronyma alchorneoides* (Phyllanthaceae)	NA	NA	NA	NA	NA	NA	49.9 ± 0.8	47.2 ± 0.2
*Macrocnemum roseum* (Rubiaceae)	NA	NA	NA	NA	NA	NA	47.3 ± 0.5	51.6 ± 0.2
*Miconia argentea* (Melastomataceae)	NA	NA	NA	NA	NA	NA	49.2 ± 1.5	48.3 ± 0.6
*Ochroma pyramidale* (Malvaceae)	NA	NA	NA	NA	NA	NA	49.3 ± 0.2	49.8 ± 1.8
*Schizolobium parahyba* (Fabaceae)^a,d^	NA	73.0	NA	26.0	NA	2.8	50.3 ± 1.0	50.9 ± 0.3
*Cecropia obtusifolia* (Urticaceae)^a,d^	NA	67.0	NA	25.0	NA	2.7	NA	48.2 ± 0.7
*Ceiba pentandra* (Malvaceae)^a,d^	NA	74.0	NA	26.0	NA	2.9	NA	45.9 ± 0.7
*Tabebuia guayacan* (Bignoniaceae)^a,d^	NA	60.0	NA	29.0	NA	2.1	NA	47.3 ± 1.9

For large trees, wood core samples were taken at 1.3 m height using a 5.15-mm-diameter increment borer. We cored only trees with straight stems that were free of heart-rot or other visible damage, and all cores were taken in directions parallel to slopes in order to avoid tension-wood biases (Du and Yamamoto 2007). For saplings, we selected individuals that were located in relatively uniform understorey light conditions (assessed by A. R. Martin) that were free of any visible damage (e.g. insect damage, chlorotic leaves, bent stems). We then used pruning shears to remove all foliar elements, and clipped the remaining stem at ∼5 cm height above ground. The middle 10-cm portion of the stem was then collected for analysis. All wood samples were placed in a freezer within 4 h of collection to avoid the loss of volatile organic compounds.

### Sample processing and laboratory analysis

All wood samples were prepared and analysed at the University of Toronto, Canada. We first used utility knives to remove bark and outer tissue that may have lost low-molecular-weight C-based volatile compounds (Thomas and Malczewski 2007; Martin and Thomas 2011), or may have been contaminated by the surface of the core borers. In our analysis, we wanted to explicitly assess ontogenetic changes in wood chemistry and, therefore, focused on only the most recently formed woody tissue. To do this, we utilized only the outer 5 cm of each large-tree core, and removed the pith from each sapling sample. Each sample was ground into a fine powder using a Wiley Mill (no. 40 mesh), and freeze-dried under a vacuum for 7 days using a Labconco 8-L freeze drying system (Labconco Co., Kansas City, MO, USA). Samples were then analysed for C using an ECS 4010 CN elemental analyser (Costech Analytical Technologies, Inc., Valencia, CA, USA) that was calibrated between each sample run using an ethylenediaminetetraacetic acid standard.

For each sample, we calculated a carbon conversion factor (C_conv_) following Martin and Thomas (2011), which expresses freeze-dried C concentration as a percentage of oven-dried mass:
(1)
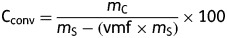

where *m*_C_ is the mass of C detected through elemental analysis, *m*_S_ is the initial mass of the sample analysed (50–60 mg) and vmf is the volatile mass fraction calculated on a separate portion of each sample as:
(2)
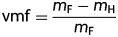

where *m*_F_ is the mass of the freeze-dried sample and *m*_H_ is the mass of the heated sample following oven drying to constant mass at 110 °C.

Values and errors of C_conv_ for each species/size class are based on analysis of three to five wood samples taken each from an individual tree, for a total of 173 observations in our dataset (Table 1) [**see Supporting Information—Table 1**].

Following the C analysis, 16 species were selected for detailed wood chemical assays. These species were chosen based on sample availability following elemental analysis, and in order to provide a broad taxonomic range (Table 1). Additionally, we selected only 16 species due to the extensive time required to perform L and H extractions (>15 h per replicate). For each group, standard protocols were used to determine H (Zobel *et al.* 1966) and L concentrations (Technical Association of Pulp and Paper Industry 2013), which are both expressed as percentages (weight/weight basis) of extractive-free wood (i.e. woody tissue comprised only H and L).

We first prepared extractive-free wood by placing samples in a soxhlet extraction apparatus with an ethanol–toluene mixture (1 L of absolute ethanol, 427 mL of toluene) for 4 h, followed by ethanol extraction for an additional 4 h. Wood samples were then air-dried, subjected to three successive 1-h extractions in 1 L of distilled water at 100 °C, and washed with 500 mL of boiling distilled water. Each sample was then oven-dried at 110 °C to constant mass and cooled in a desiccator. Each sample was then split for H and L extractions.

For each species/size class, the H extractions were performed following Zobel *et al.* (1966). First, each sample was placed in a 250-mL Erlenmeyer flask to which we added 10 mL of a solution containing 60 mL of glacial acetic acid, 20 g of NaOH, in 1 L of distilled water. Immediately thereafter, 1 mL of a 50-g NaClO_2_ in 250 mL of distilled water solution was added to each sample, which was then covered by a 50-mL Erlenmeyer flask. Flasks were then placed in a 70 ± 2 °C water bath where they were manually agitated every 30 min. An additional 1 mL of the NaClO_2_/distilled water solution was added to each flask at 45, 90 and 150 min after initial placement in the water bath. After 4 h in the bath, flasks were placed in ice-water baths and diluted with 15 mL of distilled water. Samples were then filtered using coarse-fritted crucibles, washed with two 5-mL acetone rinses, and then dried to constant weight at 110 °C. Each sample was then cooled in a desiccator and weighed, and H concentration was calculated as:
(3)
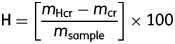

where *m*_Hcr_ is the total mass of the H sample and crucible, *m*_cr_ is the crucible mass after being cleaned, oven-dried at 110 °C to constant weight, and cooled in a desiccator, and *m*_sample_ is the mass of the extractive-free wood sample.

Lignin extractions were also performed following Technical Association of Pulp and Paper Industry (2013) standards, by adding 15 mL of 72 % sulfuric acid solution to 1 g of extractive-free wood in a beaker placed in an 18–20 °C water bath for 2 h. The concentration of sulfuric acid in each flask was then diluted to 3 % by adding 560 mL of distilled water. The mixture was then boiled for 4 h while periodically adding distilled water to maintain a volume of 560 mL. Flasks were removed from heat and, after contents settled, were filtered through a medium Gooch crucible and washed of acid with a minimum of 500 mL of hot water. Crucibles were then dried to constant mass at 110 °C, cooled in a desiccator and weighed. We then calculated L concentration as:
(4)
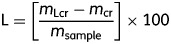



where *m*_Lcr_ is the total mass of the L sample and crucible, and *m*_cr_ and *m*_sample_ are as in Equation (3).

For each species/size group, values and errors of H and L are based on the analysis of three samples of individual trees. However, since a large amount of tissue was required for L and H analysis (i.e. 2 g of sample total), in some cases the samples we were permitted to collect (single cores) did not provide enough material once processed. Therefore, due to limited sample mass, we could only procure enough material for one to two replicates of L and H analysis in large trees of *C. triplinerve*, *P. septenatum* and *V. multiflora*. Similarly, due to sample limitations in large trees of *A. excelsum* and *P. armata*, we used published L and H values from Pettersen (1984). Published values from Pettersen (1984) are based on chemical analysis of rainforest trees in neighbouring Costa Rica (note for all species in Pettersen (1984) with species-specific records from more than one location, variation in L concentration was ≤3 %; similarly in a previous study on wood C concentration, we found intraspecific differences across sites were 2.5 % on average (Thomas and Martin 2012)).

### Statistical methods

All statistical analyses were performed using R v. 2.10.1 (R Foundation for Statistical Computing, Vienna, Austria). For each chemical trait measured, we used two-sided paired *t*-tests on species mean traits, to test for differences in wood traits at large (subscript ‘large’) vs. sapling (subscript ‘sap’) size classes. For each wood chemical trait measured, we also used linear regression to examine the relationship between the absolute size-dependent changes (calculated as the difference between sapling trait values and large-tree values) and species' life-history strategy, using WD as an indicator of life-history strategy (Chave *et al.* 2009). We also used regression analysis to quantify the relationship between wood chemical trait values at the large tree (dependent variable) and sapling (independent variable) stages.

We used principal components analysis (PCA) to examine the relationship between wood chemical traits (H, L, C_conv_) and species' relative growth rate (RGR), mortality rates (*M*), WD and maximum tree size. Since sapling-specific growth and mortality data were unavailable, this analysis was performed only on large trees. For the large trees in this analysis, growth (RGR_D10_) and mortality (M_D10_) are expressed as percentages based on trees ≥10 cm DBH during the 2000–2005 census interval at the BCI forest dynamics plot, as derived from a Bayesian hierarchical model (Condit *et al.* 2006). Maximum tree size (D95_0.1_) was calculated as the 95th quantile of diameters, for all trees ≥10 % of the maximum observed DBH (Kraft and Ackerly 2010), based on the 2010 census in the BCI 50-ha forest dynamics plot (Hubbell *et al.* 2005). Values for WD were taken from Wright *et al.* (2010), which were based on tree cores taken previously from the exact trees we then cored for H, L and C_conv_ analysis in this study.

Owing to data limitations (in terms of available functional trait and demographic data), prior to the PCA analysis we supplemented our dataset with published data where possible. To this end, we added four species for which published C_conv-large_, large-tree L, large-tree H and all life-history data were available (Table 1). For all data supplementation, large-tree L and H were taken from Pettersen (1984) and C_conv-large_ from Martin and Thomas (2011). Therefore, in total, the PCA for large trees was based on 20 species. We also used simple linear regression to evaluate bivariate relationships among wood chemical traits, and between wood chemical traits and life-history traits (see Table 1). (Note that all correlation analyses performed on raw trait values were also retested using phylogenetically independent contrasts (PICs). Phylogenetically independent contrast tests were performed following Felsenstein (1985), using the same phylogeny employed in our tests for phylogenetic signal (detailed below). Since the PIC results were largely inconclusive, they are not discussed at length here [**see Supporting Information—Table 2**].)

For all wood chemical traits, we tested for phylogenetic signal in the large-tree and sapling size classes individually, by calculating the *K* statistic in the ‘Picante’ R package (Kembel *et al.* 2010). Generally, *K* > 1 suggests that a trait has a greater phylogenetic signal than expected under Brownian motion, *K* < 1 suggests that a trait is less conserved than expected under Brownian motion, and *K* = 1 suggests that traits perfectly match a Brownian expectation (Blomberg *et al.* 2003; Kraft and Ackerly 2010). Significance values of *K* statistics were determined by randomizing traits across the phylogeny 999 times, and traits were considered to show significant phylogenetic signal if they fell within the upper 95 % of randomized *K* distributions (Blomberg *et al.* 2003; Kraft and Ackerly 2010). The phylogeny used for this analysis was based on a maximum likelihood reconstruction (D. L. Erickson *et al.*, unpubl. data) dated using three plastid loci following Kress *et al.* (2009). This phylogeny included 1347 species from 15 separate forest dynamics plots established by the Centre for Tropical Forest Science, including 337 species from the plot on BCI. The taxa for BCI were pruned from the larger phylogeny after dating with PATHd8 (Britton *et al.* 2007) and employed in current phylogenetic analyses. For each *K* test, we then used the ‘APE’ package in R (Paradis *et al.* 2004) to further prune the phylogenetic tree to include only those species that had associated wood chemical trait data.

## Results

### Inter- and intraspecific variation in wood chemical traits

**Figure 1.**
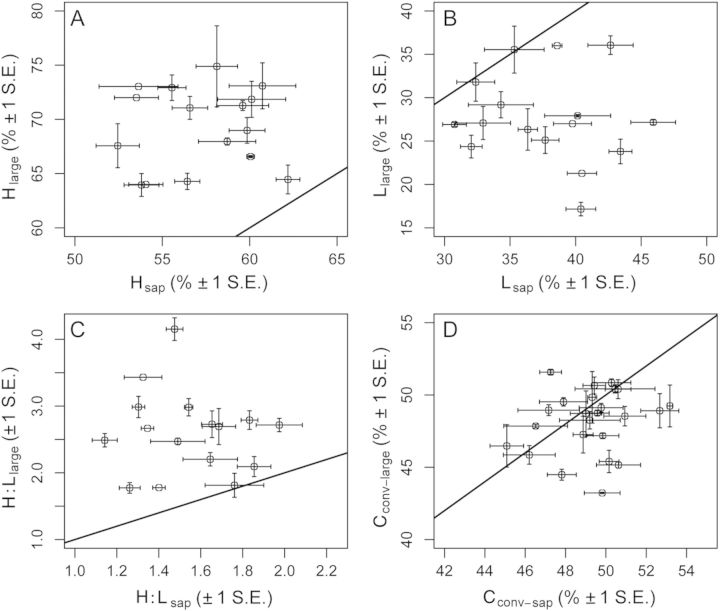
Intraspecific relationships in wood chemical traits of Panamanian tree species. Intraspecific variation in holocellulose concentration (H; A), lignin concentration (L; B) H : L ratios (C), and carbon conversion factors (C_conv_; D) for Panamanian rain forest tree species. For all four traits presented, sapling (subscript ‘sap’) and large-tree traits (subscript ‘large’) were unrelated (adj. *r*^2^ ≤ 0.0451, *P* ≥ 0.212). Error bars represent ±1 standard error of the mean (S.E.), and the solid lines represent a 1 : 1 relationship between sapling and large-tree traits.

**Figure 2.**
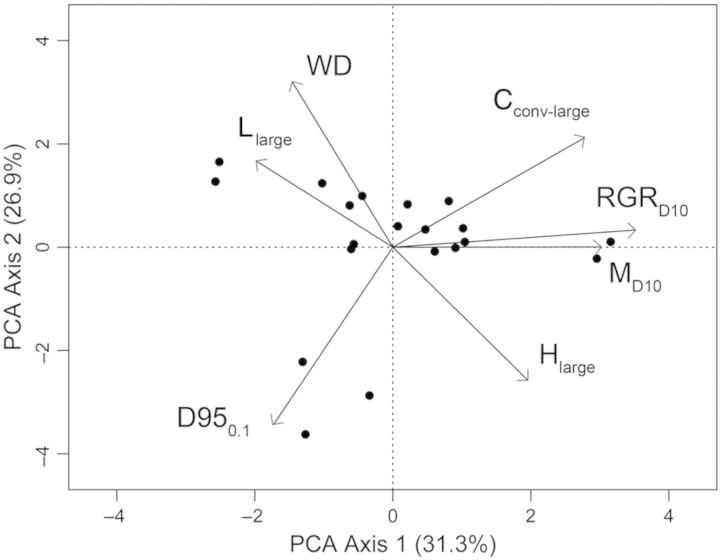
Biplots illustrating multivariate relationships between wood chemical and life-history traits for Panamanian tree species at the large-tree size class. Principal components analysis for lignin (L_large_), holocellulose (H_large_), carbon concentration (C_conv-large_), relative growth rates (RGR_D10_), mortality rate (M_D10_), WD and maximum tree size (D95_0.1_) in 20 Panamanian rainforest tree species.

All wood chemical traits tested (H, L, H: L and C_conv_) differed significantly between saplings and large trees (Table 1) [**see Supporting Information****—Figs 1 and 2**]. Among species, we observed a consistent trend whereby saplings showed lower H, lower H: L ratio, and greater L when compared with conspecific large trees (Table 1, Fig. 1) [**see Supporting Information****Figs 1 and 2**]. Similarly, in 16 of 24 species, we observed a trend whereby saplings showed greater C_conv_ compared with large trees (Table 1, Fig. 1) [**see Supporting Information**—**Figs 1 and 2**].

Across 16 species, sapling H concentration (57.2 ± 0.8 % (S.E.)) was on average 20.6 % lower than the large-tree H concentration (69.2 ± 0.9 %: one-sided paired *t*-test, *t* = −10.262, df = 15, *P* < 0.0001). This pattern was consistent in all 16 species (Fig. 1A), with the largest size-dependent increase in H concentration detected in *P. septenatum*, for which large-tree H concentration was 36 .2 % greater than sapling H concentration (Table 1) [**see Supporting Information**—**Fig. 1**]. The smallest size-dependent increase in H was detected in *V. multiflora*, for which large-tree H concentration was 3 .7 % greater than sapling H concentration (Table 1) [**see Supporting Information—Fig. 1**]. The absolute magnitude of the size-dependent change in H concentration was unrelated to WD (adj. *r*^2^ < 0, *P* = 0.704).

In the same 16 species, the concentration of L showed the opposite trend, being significantly higher in saplings when compared with large trees (one-sided paired *t*-test, *t* = 5.520, df = 15, *P* < 0.0001). Mean sapling L concentration (37.7 ± 1 .1 %) was 36 .1 % greater than mean large-tree L concentration (27.7 ± 1.3 %). With the exception of *V. multiflora*, for which sapling and large-tree L concentrations were virtually the same (35.3 ± 2.3 % and 35.5 ± 2.7 %, respectively), this trend was consistent across all species (Fig. 1B). The largest absolute size-dependent increase in L concentration was in *T. pallida*, where sapling L concentration (40.4 ± 1.1 %) was more than double (134.9 % greater) large-tree L concentration (17.2 ± 0.8 %; Table 1) [**see Supporting Information—Fig. 1]**. The absolute magnitude of the size-dependent change in L concentration was unrelated to WD (adj. *r*^2^ < 0, *P* = 0.778).

Inter- and intraspecific variations in H and L concentrations contributed to similar size-dependent patterns in H : L ratios, such that the mean H : L ratio in saplings was 69.5 % lower than that in large trees (mean sapling H : L ratio = 1.54 ± 0.1, mean large-tree H : L ratio = 2.61 ± 0.2, one-sided paired *t*-test, *t* = −6.151, df = 15, *P* < 0.0001). This pattern was consistent in all 16 species (Fig. 1C).

Compared with differences in H and L, size-dependent changes in C_conv_ for the 24 species tested here were smaller, and also less consistent. In absolute terms, mean C_conv-sap_ (49.2 ± 0.4 %) was only marginally (and not significantly) higher than C_conv-large_, by 1 % on average (48.2 ± 0 .4 %, two-sided paired *t*-test, *t* = 1.952, df = 23, *P* = 0.063). This pattern was observed in 16 of 24 species (Fig. 1, Table 1) [**see Supporting Information—Fig. 2**]. The largest size-dependent decrease in C_conv_ was found in *Guarea* ‘fuzzy’ (6.6 % reduction in C_conv-large_ compared with C_conv-sap_), while *M. roseum* showed the largest size-dependent increase in C_conv_ at 4.3 % (Table 1) [**see Supporting Information—Fig. 2]**. The absolute magnitude of size-dependent changes in C_conv_ was unrelated to WD (adj. *r*^2^ < 0, *P* = 0.585).

We found that wood chemical traits at larger tree sizes were not predicted by sapling values (adj. *r*^2^ < 0, *P* = 0.40–0.79; Fig. 1, Table 2).

**Table 2.** Relationships between wood chemical traits and life-history traits in Panamanian tree species. Coefficients of determination (adj. *r*^2^), associated *P*-values (in parentheses), and sample sizes for pairwise correlation analyses between wood chemical traits and life-history traits (for large trees) for Panamanian tree species. Wood chemical traits tested are holocellulose (H), lignin (L), H : L ratios and carbon (C_conv_) concentrations for the sapling (subscript ‘sap’) and large-tree (subscript ‘large’) size classes. Life-history traits tested are WD, maximum tree size (D95_0.1_) and mortality rates for trees ≥10 cm DBH (M_D10_ and RGR_D10_, respectively). Statistically significant relationships (*P* < 0.05) are highlighted in bold.

	H_sap_	L_sap_	C_conv-sap_	H_large_	L_large_	C_conv-large_
L_sap_	**0.287 (0.019), *n* = 16**	–				
C_conv-sap_	0.007 (0.311), *n* = 16	0.165 (0.067) *n* = 16	–			
H_large_	0.000 (0.792), *n* = 16	NA	NA	–		
L_large_	NA	0.000 (0.663) *n* = 16	NA	**0.364 (0.003),** *n* = 20	–	
C_conv-large_	NA	NA	0.000 (0.4), *n* = 24	0.023 (0.244), *n* = 20	0.042 (0.195), *n* = 20	–
WD	NA	NA	NA	0.054 (0.167), *n* = 20	0.000 (0.718), *n* = 20	0.000 (0.984), *n* = 27
D95_0.1_	NA	NA	NA	0.007 (0.303), *n* = 20	0.000 (0.787), *n* = 20	0.04 (0.166), *n* = 26
M_D10_	NA	NA	NA	0.000 (0.764), *n* = 20	0.000 (0.854), *n* = 20	**0.146 (0.031),** *n* = 26
RGR_D10_	NA	NA	NA	0.000 (0.545), *n* = 20	0.000 (0.879), *n* = 20	0.000 (0.348), *n* = 26

### Relationships among wood chemical and life-history traits

A visualization of the correlations among wood chemical and species' life-history traits in large trees is presented in Fig. 2. The first PCA explained 31.3 % of the variation in traits and was strongly associated with species' demographic traits (RGR_D10_: adj. *r*^2^ = 0.626, *P* < 0.0001, M_D10_: adj. *r*^2^ = 0.443, *P* = 0.001), and C_conv-large_ (adj. *r*^2^ = 0.369, *P* = 0.003). PCA axis 1 was also significantly, though weakly, associated with large-tree H concentration (adj. *r*^2^ = 0.157, *P* = 0.047) and large-tree L concentration (adj. *r*^2^ = 0.159, *P* = 0.046). The second PCA axis explained 26.9 % of the variation among species and was strongly associated with D95_0.1_ (adj. *r*^2^ = 0.596, *P* < 0.001) and WD (adj. *r*^2^ = 0.508, *P* < 0.001). PCA axis 2 was also correlated with C_conv-large_ (adj. *r*^2^ = 0.192, *P* = 0.031) and large-tree H (adj. *r*^2^ = 0.312, *P* = 0.006), and weakly correlated with large-tree L (adj. *r*^2^ = 0.097, *P* = 0.098). PCA axis 2 was unrelated to RGR_D10_ and M_D10_ (adj. *r*^2^ < 0 and *P* ≥ 0.975 in both cases).

Bivariate trait relationships in our dataset were generally weak (Table 2). The only trait correlation consistent at both sizes was between L and H, which were significantly negatively correlated in both the sapling (adj. *r*^2^ = 0.287, *P* = 0.019) and large-tree size classes (adj. *r*^2^ = 0.193, *P* = 0.021, Table 2). The only significant relationship between wood chemical traits and life-history traits we observed was between C_conv-large_ and M_D10_ (adj. *r*^2^ = 0.146, *P* = 0.031, Table 2). When analysed in a phylogenetic framework using PICs, however, none of these correlations were significant [**see Supporting Information—Table 2**].

### Phylogenetic signal in wood chemical traits

In the large-tree size class, we only found evidence for significant phylogenetic signal in H concentration (*K* = 0.321, *P* = 0.009). Of all traits tested at either size class, large-tree L and H : L ratio showed the next largest *K* values (*K* = 0.119 and 0.096, respectively), but neither was significantly different from a null expectation (*P* ≥ 0.25). We did not detect phylogenetic signal in C_conv__-large_ (*K* = 0.062, *P* = 0.419). At the sapling stage, there was no evidence for phylogenetic signal in any of the four wood chemical traits tested here (*K* ≤ 0.054, *P* ≥ 0.435; Table 3).

**Table 3.** Phylogenetic signal in wood chemical traits of Panamanian trees. Results of tests for phylogenetic signal in four wood chemical traits at the sapling and large-tree size classes. Traits showing significant phylogenetic signal are highlighted in bold typeface.

Trait	Saplings			Large trees		
	*n*	*K*	*P*	*n*	*K*	*P*
Holocellulose concentration (H)	16	0.054	0.435	20	0.323	**0**.**009**
Lignin concentration (L)	16	0.032	0.679	20	0.119	0.182
H : L ratio	16	0.04	0.625	20	0.096	0.25
Carbon concentration (C_conv_)	24	0.038	0.716	27	0.062	0.419

## Discussion

We found that all wood chemical traits tested here varied significantly with tree size (Table 1, Fig. 1) [**see Supporting Information—Figs 1 and 2**]. Our hypothesis regarding the direction of size-dependent changes in wood chemical traits was strongly supported: in nearly all species, saplings had higher L concentration compared with conspecific large trees, and in all species saplings had both lower H concentration and H : L ratio when compared with conspecific large trees (Fig. 1A–C) [**see Supporting Information—Fig. 1**]. Our hypothesis about the direction of size-dependent change in wood C concentration was also largely supported. In more than 60 % of species tested (16 of 24), sapling wood contained more C than conspecific large trees (Fig. 1D) [**see Supporting Information—Fig. 2**]. Wood chemical traits in large trees could not be predicted by wood chemical traits in saplings (Fig. 1, Table 2). Multivariate and correlation analyses suggest that L and H concentrations in large trees are not strongly associated with demographic or other functional traits tested here (Fig. 2, Table 2). Lastly, our prediction regarding phylogenetic signal in wood chemical traits was not well supported. In large trees only the concentration of H showed a significant phylogenetic signal, whereas all sapling wood chemical traits, as well as wood C, L and H : L in large trees, did not show a significant phylogenetic signal (Table 3).

### Intraspecific variation in wood chemistry

In the 16 species for which sapling and large-tree L data were collected, not only was the mean sapling L concentration (37.7 %) 9.8 % greater than the mean large-tree L concentration (27.9 %), but the mean sapling L concentration was also larger than the maximum observed large-tree L concentration (36.1 % in *P. armata*, Fig. 1B). This finding is remarkably consistent when comparing sapling L concentration in Panamanian species with L concentrations found in large trees from several tropical forest sites. Specifically, mean sapling L concentrations found here were greater than, or approximately equal to, maximum L concentrations found in large trees of over 80 species from African, South American and Southeast Asian forests (Nuopponen *et al.* 2006; Santana and Okino 2007; Rana *et al.* 2010; van Geffen *et al.* 2010). Because our values for large trees were comparable with these published accounts (e.g. mean large-tree L of 31.6 % in Santana and Okino 2007), the remarkably high values for sapling L concentrations reported here cannot be attributed to methodological inconsistencies.

Why saplings favour higher allocation to L appears to be well explained by a combination of (i) a need for defence from pathogens and (ii) developmental constraints associated with biomechanical stability. With respect to pathogens, studies have inferred the defensive role of L in wood based on a negative relationship between L and wood decomposition rates (Scheffer and Cowling 1966; Cornwell *et al.* 2009; van Geffen *et al.* 2010). This assumption is best supported by early experimental work by Pew (1957) who found that grinding wood (i.e. breaking the ligno-cellulosic structures) renders tissue more susceptible to enzymatic decay. Recent work has shown common pathogens can affect multiple hosts, but have unequal effects on mortality rates across species (Augspurger and Wilkinson 2007; Hersh *et al.* 2012). These unequal effects are believed to be a function of (i) the aggressiveness of a pathogen as dictated by environmental conditions and pathogen–pathogen interactions (Augspurger 1984; Alvarez-Loayza *et al.* 2011; Hersh *et al.* 2012), (ii) physiology of the host tree species including its shade tolerance or ability to compartmentalize fungal attacks (McCarthy-Neumann and Kobe 2008; Schmidt 2010) or (iii) the phylogenetic structure of the local tree species community (Gilbert and Webb 2007). Explicitly testing pathogen susceptibility as a function of L concentration, or comparing wood L concentrations in conspecifics grown in high- vs. low-pathogen environments, would help determine the importance of pathogens as drivers of size-dependent (and interspecific) variation in L concentration.

Support for the ‘mechanical stability’ explanation is less clear. Although the influence of L on wood mechanical stiffness is considered well established (e.g. Whetten and Sederoff 1995), explicit tests relating wood chemical traits to mechanical traits (i.e. modulus of rupture, modulus of elasticity (MOE), compressive strength, etc.) in live hardwoods are few. Experimental studies of dried wood used as a construction material indicate that delignification has little or no effect on mechanical properties, but drastically reduces wood mechanical resistance to water exposure (Winandy and Rowell 2005); however, the relevance of this work to live wood is unclear. Additionally, mechanical strength at a whole-stem level is a function of how woody tissues both resist and displace stress, with the relative importance of these mechanisms being dictated by the structural arrangement of chemical constituents within cell walls (Lucas *et al.* 2000). For this reason, explicitly quantifying the role of L in conferring mechanical stability would need to control for variation in the intracellular structure of cell walls. Lastly, to quantify the relative influence of chemical vs. structural traits in determining realized tree mechanical stability, one must also account for possible radial variation in wood chemical traits. Radial variation in WD has been well documented in tropical trees (Wiemann and Williamson 1988; Nock *et al.* 2009). But nearly all existing knowledge on radial variation in wood chemistry is based on patterns observed in temperate conifers, and generalized from categorical comparisons between heartwood vs. sapwood, not accounting for large intermediary zones in tree stems (Lachenbruch *et al.* 2011).

### Carbon concentration in saplings and large trees

It is reasonable to assume that size-dependent declines in L might also result in size-dependent declines in C_conv_ (Lamlom and Savidge 2003). Our data, however, do not support this explanation given the lack of significant relationships between C_conv-large_ and large-tree L concentration (Table 2), and a weak relationship between C_conv-sap_ and sapling L concentration (Table 2). Moreover, assuming a constant C concentration for L and H molecules, a mean size-dependent decline of 9.8 % in L concentration corresponds to a size-dependent reduction in C_conv_ of 1. 96 %: nearly twice as large as our observed size-dependent decrease in C_conv_ of 1 %. Therefore, we suggest that it is likely that in maturing from saplings to large trees, declines in L are offset by increases in other C-based compounds in wood such as volatile C compounds. In our species, these low-molecular-weight compounds—including terpenoids, phenolics and alcohols—comprise on average 2.5 % (weight/oven-dried weight) of wood at the large-tree sizes (Martin and Thomas 2011). Although we did not explicitly quantify volatile C in saplings, volatile mass fraction in saplings (vmf, see Equation 2) was negligible (mean vmf = 0.000021 ± 0.00001, range = 0–0.0003, *n* = 24 species). Hence, we speculate that the modest size-dependent declines in C_conv_ in our data may reflect a shift in allocation from L to secondary volatile C compounds, which may also play a role in plant defence.

In terms of size dependence of C_conv_, our results suggest the inverse of IPCC (2006) carbon accounting protocols that assume tropical large-tree wood is more carbon rich (49 % C) than small-tree wood (46 % C) (Fig. 1D) [**see** **Supporting Information****—Fig. 2**]. Although our dataset shows only a modest size-dependent decrease in C_conv_ with overlapping 95 % confidence intervals, this difference is larger when comparing C_conv-sap_ with larger tropical wood C datasets (i.e. mean C_conv-large_ of 47.4 ± 0.33 % (S.E.) in 59 Panamanian species studied by Martin and Thomas (2011)). The degree to which sapling-specific wood C will influence forest C accounting is likely small, as trees ≤10 cm DBH generally comprise <5 % of total aboveground biomass in mature tropical forests (e.g. Clark *et al.* 2001). Nevertheless, size-dependent changes in C_conv_ should be taken into account in secondary forests and recently reforested areas, where small trees constitute a larger proportion of total forest biomass (Preece *et al.* 2012).

### Ecological correlates of wood chemical traits

With the sole exception of a significant relationship between C_conv_ and mortality, wood chemical traits in large trees were neither correlated with species' demographic rates nor ecological traits in our dataset (Table 2). This is consistent with existing studies testing relationships between wood C concentration and life-history traits in >10 temperate or tropical species (Thomas and Malczewski 2007; Martin and Thomas 2011).

In multivariate space, large-tree WD was positively associated with the L concentration and negatively associated with the H concentration (Fig. 2). Although these relationships were not significant when tested in a bivariate framework, these results could indicate that wood anatomical and chemical traits are correlated in tropical trees, when larger numbers of species are available for analysis. Current evidence for the generality and direction of these potential relationships remains mixed. For example, a recent meta-analysis found that L concentration and WD are negatively correlated in angiosperms (Weedon *et al.* 2009), and a study using transgenic *Populus* trees demonstrated that L concentration and WD can vary independently, and that greater wood stiffness associated with higher L concentration can occur with little change in WD (Voelker *et al.* 2011). These inconsistencies point out the need for evaluating more species when testing relationships between wood chemical and anatomical traits.

### Phylogenetic signal in wood chemical traits

In a previous study, we did not detect a phylogenetic signal in wood C in large tropical trees (Martin and Thomas 2011). Here we found evidence for a phylogenetic signal in large-tree H, but not in L concentrations or in C_conv-large_ (Table 3). Although the *K* value for large-tree L was not significant compared with a randomized *K* distribution (*K* = 0.119, *P* = 0.182), large-tree L was considerably greater than all other observed *K* values (Table 3). Basic wood chemical constituents (H and L) might be expected to show greater phylogenetic signal when compared with C_conv_, since C_conv_ integrates several compounds such as C-based secondary chemicals (Lamlom and Savidge 2003; Thomas and Malczewski 2007; Martin and Thomas 2011) and non-structural carbohydrates that can be sensitive to short-term changes in growing conditions such as light or rainfall (Newell *et al.* 2002; Veneklaas and den Ouden 2005). Site-specific and seasonal variabilities of these compounds in wood may further obscure the phylogenetic signal in C_conv_. Stem H and L concentrations may also vary as a function of growing conditions, but current literature focuses mainly on changes in wood H and L concentrations as a response to stem damage (Whetten and Sederoff 1995; Guariguata and Gilbert 1996) and/or reaction wood formation (Gindl 2002; Kibblewhite *et al.* 2010). Here we attempted to control for these factors by selecting straight trees free of mechanical damage.

A non-significant phylogenetic signal and considerably lower *K* values for sapling chemical traits compared with large trees is consistent with developmental constraints driving only small differences in sapling wood chemistry. Among the 16 species examined, we observed a more restricted range for sapling L and H concentrations (15.2 and 10.0 %, respectively) when compared with large-tree L and H concentrations (18.9 and 10.9 %, respectively, Fig. 1). We speculate that these similarities among distantly related taxa are due to shared, unavoidable requirements for pathogen resistance, and potentially biomechanical stability. The explanation of convergence in wood chemical traits due to a need for resistance to cellulose-degrading pathogens is particularly appealing, given the wide taxonomic range of hosts infected by many common pathogens (Augspurger and Wilkinson 2007; Hersh *et al.* 2012). Investing in broadly effective constitutive defences such as L may be a useful defensive strategy for seedlings and saplings from a wide range of taxonomic groups.

## Conclusions

Our dataset indicates consistent size-dependent changes in wood chemical traits in tropical trees. Whether this pattern can be generalized to other tree species and sites outside of Panama, however, requires further testing. Our results were consistent with the hypothesis that size-dependent changes in wood chemical traits are adaptive responses to changes in environmental conditions and biotic stresses experienced by trees during the course of their development. Further exploration of wood chemical traits in relation to individual tree performance, such as survivorship in the forest understorey, would greatly enhance our understanding of the functional significance of wood chemical traits. Examining variation in species-specific wood chemical traits along a size continuum, and linking chemical traits directly to tree performance, would also contribute significantly towards a comprehensive understanding of the functional ecology of wood traits in both tropical and temperate trees.

## Sources of Funding

The HSBC Climate Partnership, the Natural Sciences and Engineering Research Council of Canada, and both the Jeanne F. Goulding Fellowship program and the Centre for Global Change Science at the University of Toronto generously provided funding for this study.

## Contributions by the Authors

A.R.M. conceived and designed the study, performed field data collection, conducted both lab and statistical analyses and wrote the manuscript. S.C.T. conceived and designed the study and wrote the manuscript. Y.Z. performed laboratory analysis and wrote the manuscript.

## Conflicts of Interest Statement

None declared.

## Supporting information

The following additional information is available in the online version of this article—

**Table 1.** Taxonomy, species codes and sample sizes used to determined holocellulose concentration (H), lignin concentration (L), H : L ratios (H : L) and carbon concentration (C_conv_) values for Panamanian rainforest tree species. Sample sizes are given for determinations at the sapling (subscript ‘sap’) and large-tree (subscript ‘large’) size classes. Superscripts following species names refer to: ^1^H_large_, L_large_ and H : L_large_ data from Pettersen (1984), ^2^C_conv-large_ data taken from Martin and Thomas (2011).

**Table 2.** Phylogenetically independent contrasts (PIC) between wood chemical traits and life-history traits in Panamanian tree species. Coefficients of determination (adj. *r*^2^), associated *P*-values (in brackets), and number of species used in PIC analysis between wood chemical traits and life-history traits (for large trees) for Panamanian tree species. PIC analyses were performed following Felsenstein (1985). Wood chemical traits tested are holocellulose (H), lignin (L), H : L ratios, and carbon (C_conv_) concentrations for the sapling (subscript sap) and large tree (subscript large) size classes. Life-history traits tested are wood density (WD), maximum tree size (D95_0.1_), and mortality rates for trees ≥ 10 cm DBH (M_D10_ and RGR_D10_, respectively).

**Fig. 1.** Size-dependent variation in wood chemical traits of 16 Panamanian tree species. Mean holocellulose concentration (H, Panel A), lignin concentration (L, Panel B), and H : L ratios (Panel C) in woody tissues of 16 Panamanian species at sapling (dark bars) and large tree (light bars) size classes. Error bars represent ± 1 standard error of the mean (S.E.). Species codes correspond to Supplementary Table 1, and the phylogenetic tree represents evolutionary relationships among species based on a maximum likelihood reconstruction (D. L. Erickson *et al.*, unpubl. data) dated using three plastid loci following Kress *et al.* (2009).

**Fig. 2.** Size-dependent variation in wood carbon content in 24 Panamanian tree species. Mean carbon conversion factors (C_conv_) in woody tissues of 24 Panamanian species at sapling (dark bars) and large tree (light bars) size classes. Error bars represent ± 1 standard error of the mean (S.E.). Species codes correspond to Supplementary Table 1, and the phylogenetic tree represents evolutionary relationships among species based on a maximum likelihood reconstruction (D. L. Erickson *et al.*, unpubl. data) dated using three plastid loci following Kress *et al.* (2009).

Additional Information
