# Supplementary material for: Size-dependent changes in wood chemical traits: a comparison of neotropical saplings and large trees
Source: AoB Plants. 2013 Aug 29;5:plt039. doi: 10.1093/aobpla/plt039 (PMC4455665; doi:10.1093/aobpla/plt039)
Supplement: Additional Information [file supp_5_plt039_index.html]

Size-dependent changes in wood chemical traits: a comparison of neotropical saplings and large trees — Additional Information 

# Size-dependent changes in wood chemical traits: a comparison of neotropical saplings and large trees

## Additional Information

Additional Information

**Files in this Data Supplement:**

- Additional Information Figure 1 - tif file
- Additional Information Figure 2 - tif file
- Additional Information 1 - doc file
- Additional Information 2 - doc file
