# Supplementary figures and images for: Size-dependent changes in wood chemical traits: a comparison of neotropical saplings and large trees
Source: AoB Plants. 2013 Aug 29;5:plt039. doi: 10.1093/aobpla/plt039 (PMC4455665; doi:10.1093/aobpla/plt039)

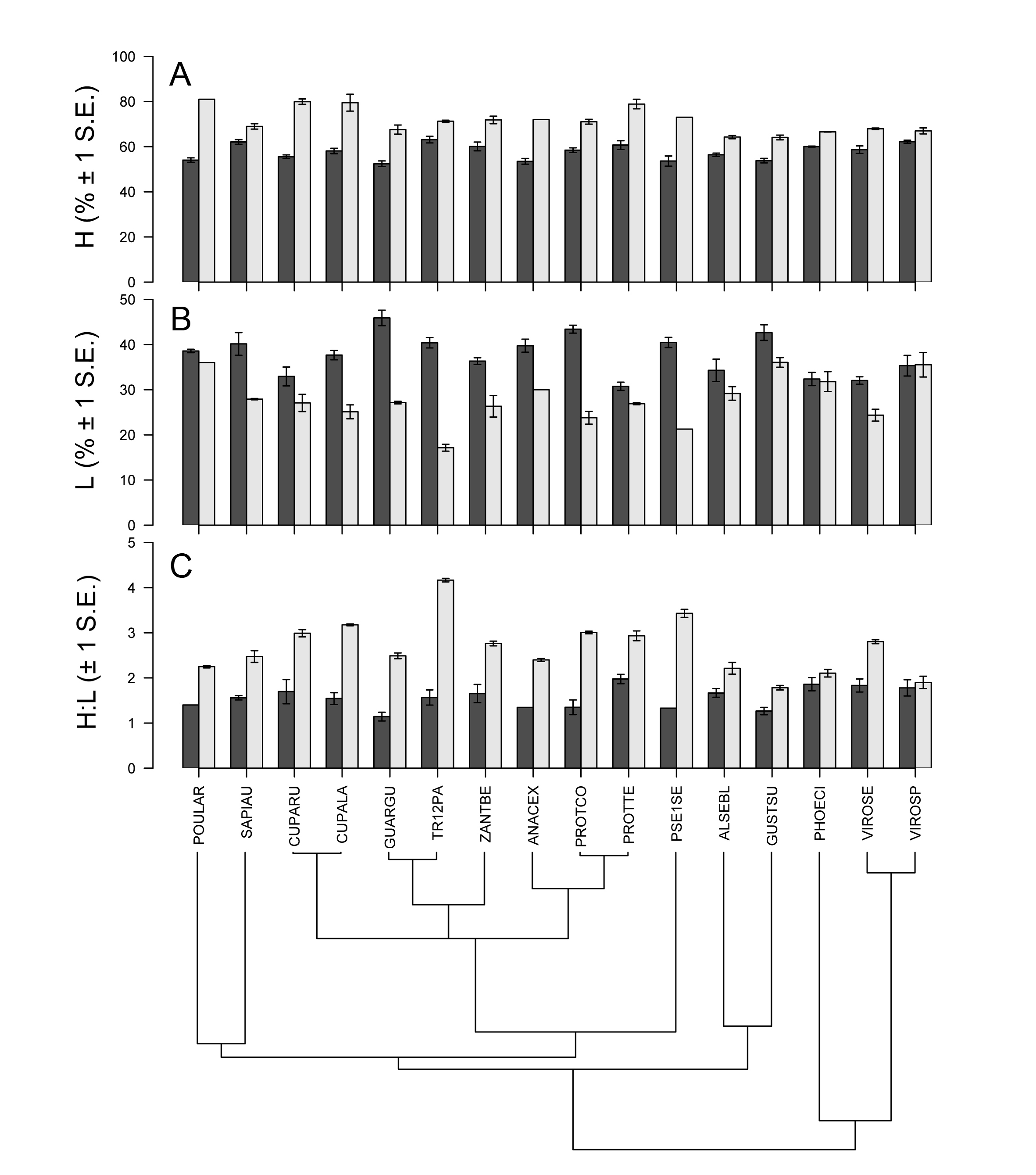

Supplement: Additional Information [file supp_plt039_plt039supp_fig1.tif]

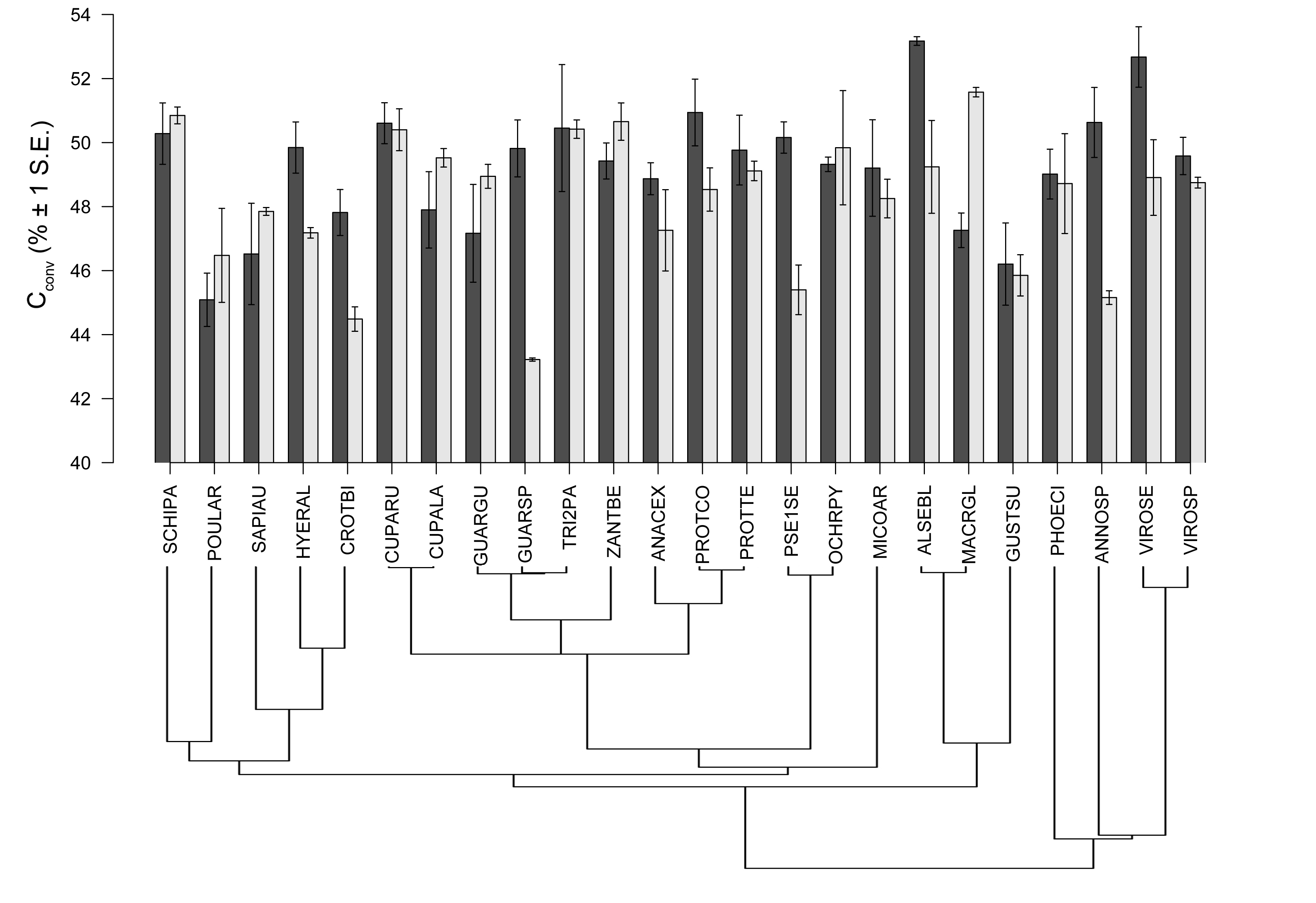

Supplement: Additional Information [file supp_plt039_plt039supp_fig2.tif]
