# Supplementary material for: Size-dependent changes in wood chemical traits: a comparison of neotropical saplings and large trees
Source: AoB Plants. 2013 Aug 29;5:plt039. doi: 10.1093/aobpla/plt039 (PMC4455665; doi:10.1093/aobpla/plt039)
Supplement: Additional Information [file supp_plt039_plt039supp_table1.doc]

**Supplementary Table 1**

Taxonomy, species codes, and sample sizes used to determined holocellulose concentration (H), lignin concentration (L), H: L ratios (H:L), and carbon concentration (C_conv_) values for Panamanian rainforest tree species. Sample sizes are given for determinations at the sapling (subscript “sap”) and large tree (subscript “large”) size classes. Superscripts following species names refer to: ^1^ H_large_, L_large_ and H:L_large_ data from Pettersen (1984), ^2^ C_conv-large_ data taken from Martin and Thomas (2011).

|  |  | Holocellulose (H) | | Lignin (L) | | Holocellulose: Lignin (H:L) | | Carbon (C_conv_) | |
| --- | --- | --- | --- | --- | --- | --- | --- | --- | --- |
| Species | Species | H_sap_ | H_large_ | L_sap_ | L_large_ | H:L_sap_ | H:L_large_ | C_conv-sap_ | C_conv-large_ |
| *Alseis blackiana*  (Rubiaceae) | ALSEBL | 3 | 3 | 3 | 3 | 3 | 3 | 4 | 4 |
| *Anacardium excelsum*  (Anacardiaceae)^1^ | ANACEX | 3 | 1 | 3 | 1 | 3 | 1 | 4 | 5 |
| *Cinnamomum triplinerve*  (Lauraceae) | PHOECI | 3 | 2 | 3 | 2 | 3 | 2 | 4 | 3 |
| *Cupania latifolia*  (Sapindaceae) | CUPALA | 3 | 3 | 3 | 3 | 3 | 3 | 4 | 3 |
| *Cupania rufescens*  (Sapindaceae) | CUPARU | 3 | 3 | 3 | 3 | 3 | 3 | 4 | 3 |
| *Guarea guidonia*  (Meliaceae) | GUARGU | 3 | 3 | 3 | 3 | 3 | 3 | 4 | 3 |
| *Gustavia superba*  (Lecythidaceae) | GUSTSU | 3 | 3 | 3 | 3 | 3 | 3 | 3 | 5 |
| *Poulsenia armata*  (Moraceae)^1^ | POULAR | 3 | 1 | 3 | 1 | 3 | 1 | 4 | 3 |
| *Protium costaricense*  (Burseraceae) | PROTCO | 3 | 3 | 3 | 3 | 3 | 3 | 4 | 3 |
| *Protium tenuifolium*  (Burseraceae) | PROTTE | 3 | 3 | 3 | 3 | 3 | 3 | 4 | 3 |
| *Pseudobombax septenatum*  (Malvaceae) | PSE1SE | 3 | 1 | 3 | 1 | 3 | 1 | 4 | 3 |
| *Sapium glandulosum*  (Euphorbiaceae) | SAPIAU | 3 | 3 | 3 | 3 | 3 | 3 | 4 | 3 |
| *Trichilia pallida*  (Meliaceae) | TRI2PA | 3 | 3 | 3 | 3 | 3 | 3 | 4 | 3 |
| *Virola multiflora*  (Myristicaceae) | VIROSP | 3 | 2 | 3 | 2 | 3 | 2 | 4 | 3 |
| *Virola sebifera*  (Myristicaceae) | VIROSE | 3 | 3 | 3 | 3 | 3 | 3 | 4 | 3 |
| *Zanthoxylum ekmanii*  (Rutaceae) | ZANTBE | 3 | 3 | 3 | 3 | 3 | 3 | 4 | 3 |
| *Annona spraguei*  (Annonaceae) | ANNOSP | NA | NA | NA | NA | NA | NA | 4 | 4 |
| *Croton billbergianus*  (Euphorbiaceae) | CROTBI | NA | NA | NA | NA | NA | NA | 4 | 3 |
| *Guarea "fuzzy"*  (Meliaceae) | GUARSP | NA | NA | NA | NA | NA | NA | 4 | 3 |
| *Hieronyma alchorneoides*  (Phyllanthaceae) | HYERAL | NA | NA | NA | NA | NA | NA | 4 | 3 |
| *Macrocnemum roseum*  (Rubiaceae) | MACRGL | NA | NA | NA | NA | NA | NA | 2 | 4 |
| *Miconia argentea*  (Melastomataceae) | MICOAR | NA | NA | NA | NA | NA | NA | 4 | 3 |
| *Ochroma pyramidale*  (Malvaceae) | OCHRPY | NA | NA | NA | NA | NA | NA | 4 | 3 |
| *Schizolobium parahyba*  (Fabaceae)^1, 2^ | SCHIPA | NA | 1 | NA | 1 | NA | 1 | 4 | 4 |
| *Cecropia obtusifolia*  (Cecropiaceae)^1, 2^ | CECROB | NA | 1 | NA | 1 | NA | 1 | NA | 3 |
| *Ceiba pentandra*  (Bombacaceae)^1, 2^ | CEIBPE | NA | 1 | NA | 1 | NA | 1 | NA | 4 |
| *Tabebuia guayacan*  (Bignoniaceae)^1, 2^ | TAB1GU | NA | 1 | NA | 1 | NA | 1 | NA | 5 |
