# Supplementary material for: Size-dependent changes in wood chemical traits: a comparison of neotropical saplings and large trees
Source: AoB Plants. 2013 Aug 29;5:plt039. doi: 10.1093/aobpla/plt039 (PMC4455665; doi:10.1093/aobpla/plt039)
Supplement: Additional Information [file supp_plt039_plt039supp_table2.doc]

**Supplementary Table 2.** Phylogenetically independent contrasts (PIC) between wood chemical traits and life-history traits in Panamanian tree species.

Coefficients of determination (adj. *r*^2^), associated *P*-values (in brackets), and number of species used in PIC analysis between wood chemical traits and life-history traits (for large trees) for Panamanian tree species. PIC analyses were performed following Felsenstein (1985). Wood chemical traits tested are holocellulose (H), lignin (L), H: L ratios, and carbon (C_conv_) concentrations for the sapling (subscript “sap”) and large tree (subscript “large”) size classes. Life-history traits tested are wood density (WD), maximum tree size (D95_0.1_), and mortality rates for trees ≥ 10 cm DBH (M_D10_ and RGR_D10_, respectively).

|  | H_sap_ | L_sap_ | C_conv-sap_ | H_large_ | L_large_ | C_conv-large_ |
| --- | --- | --- | --- | --- | --- | --- |
| L_sap_ | 0.000 (0.43)  *n* = 16 | - |  |  |  |  |
| C_conv-sap_ | 0.093 (0.133)  *n* = 16 | 0.134 (0.09)  *n* = 16 | - |  |  |  |
| H_large_ | **0.647 (0.0001)**  *n* = 16 | NA | NA | - |  |  |
| L_large_ | NA | 0.012 (0.297)  *n* = 16 | NA | **0.24 (0.002)**  *n* = 20 | - |  |
| C_conv-large_ | NA | NA | 0.000 (0.911)  *n* = 24 | 0.000 (0.709)  *n* = 20 | 0.000 (0.926)  *n* = 20 | - |
| WD | NA | NA | NA | 0.000 (0.468)  *n* = 20 | 0.061 (0.153)  *n* = 20 | 0.05 (0.138)  *n* = 27 |
| D95_0.1_ | NA | NA | NA | 0.061 (0.152)  *n* = 20 | 0.016 (0.269)  *n* = 20 | **0.26 (0.005)**  *n* = 26 |
| M_D10_ | NA | NA | NA | **0.189 (0.032)**  *n* = 20 | 0.000 (0.6)  *n* = 20 | 0.000 (0.609)  *n* = 26 |
| RGR_D10_ | NA | NA | NA | **0.188 (0.032)**  *n* = 20 | 0.058 (0.159)  *n* = 20 | **0.175 (0.019)**  *n* = 26 |
